# Detection of the Bacterium, *Xylella fastidiosa*, in Saliva of Glassy-Winged Sharpshooter, *Homalodisca vitripennis*

**DOI:** 10.1673/031.008.3401

**Published:** 2008-04-29

**Authors:** Jose L. Ramirez, Paulo T. Lacava, Thomas A. Miller

**Affiliations:** ^1^ Department of Entomology, University of California Riverside, USA; ^2^ Department of Molecular Microbiology and Immunology, Johns Hopkins Bloomberg School of Public Health, Baltimore, MD, USA; ^3^ Departamento de Genética, Escola Superior de Agricultura “Luiz de Queiroz,” Universidade de São Paulo, Piracicaba, SP, Brazil

**Keywords:** *H*. *vitripennis* probing, sharpshooter feeding, plant pathogen transmission

## Abstract

*Homalodisca vitripennis* (Germar) (Hemiptera: Cicadellidae), the glassy-winged sharpshooter, is one of the most important vectors of the bacterium, *Xylella fastidiosa* subsp. *piercei* (Xanthomonadales: Xanthomonadaceae) that causes Pierce's Disease in grapevines in California. In the present study we report a new method for studying pathogen transmission or probing behavior of *H*. *vitripennis*. When confined, *H*. *vitripennis* attempt to probe the surface of sterile containers 48 hours post-acquisition of *X*. *f*. *piercei*. The saliva deposited during attempted feeding probes was found to contain *X*. *f*. *piercei*. We observed no correlation between *X*. *f*. *piercei* titers in the foregut of *H*. *vitripennis* that fed on *Xylella*-*infected* grapevines and the presence of this bacterium in the deposited saliva. The infection rate after a 48 h post-acquisition feeding on healthy citrus and grapevines was observed to be 77% for *H*. *vitripennis* that fed on grapevines and 81% for *H*. *vitripennis* that fed on citrus, with no difference in the number of positive probing sites from *H*. *vitripennis* that fed on either grapevine or citrus. This method is amenable for individual assessment of *X*. *f*. *piercei*-infecuvity, with samples less likely to be affected by tissue contamination that is usually present in whole body extracts.

## Introduction

The glassy-winged sharpshooter, *Homalodisca vitripennis* (Germar) (Hemiptera: Cicadellidae) [formerly *H*. *coagulata* (Takiya et al. 2006)], has become a pest in California due to its ability to transmit pathogens causing scorch diseases in a number of host plants including *Xylella fastidiosa* subsp. *piercei* (Xanthomonadales: Xanthomonadaceae) that causes Pierce's Disease in grapevines (Purcell 2005). *H*. *vitripennis* is a xylophagous insect that feeds on hundreds of plant species (Purcell and Hopkins 1996; Purcell and Saunders 1999); citrus is one of its preferred hosts (Blua et al. 1999). Perring et al. (2001) demonstrated a relationship between Pierce's Disease incidence in grapes and the proximity of vineyards to citrus orchards. Furthermore, *X*. *f*. *piercei* has been shown to survive in citrus xylem but to form clumps and irregular biofilms (Toscano et al. 2004).

*X*. *f*. *piercei* uses fimbriae to attach itself to its host plants and inside the foregut of its vectors (Newman et al. 2004). Biofilm formations *of X*. *f*. *piercei* inside the precibarium of the sharpshooter is reported to be necessary for efficient transmission (Newman et al. 2004). It has been hypothesized that *X*. *f*. *piercei* cells dislodge from biofilms in the precibarium during specific probing behaviors allowing *X*. *f*. *piercei* to be inoculated into the xylem vessels (Almeida et al. 2005b). Furthermore, probing behavior has been implicated as an important factor for successful inoculation given that *H*. *vitripennis* can transmit *X*. *f*. *piercei* to plants under negative or positive pressure (Almeida et al. 2005a).

When placed in sterile plastic or glass containers, *H*. *vitripennis* nymphs or adults will press their labial tips against the surface and attempt to penetrate the surface with sawing mandibular stylets while exuding saliva droplets. This process has been previously described in great detail by Backus et al. (2005). The penetrations actually leave scratches in plastic Petri dish surfaces. When analyzed by PCR, these salivary deposits contain the pathogen, *X*. *f*. *piercei*. We report here the effect of two hosts plant, citrus and grapevines, on the presence of *X*. *f*. *piercei* in *H*. *vitripennis* saliva.

## Materials and Methods

**Figure 1.**
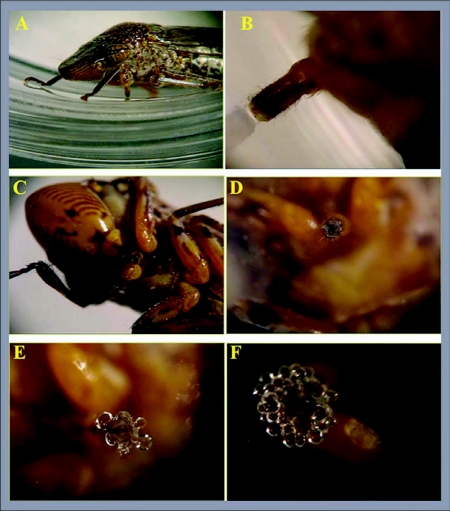
Probing session sequence and deposition of saliva. A. Glassy-winged sharpshooter in microcentrifuge tube, B. Labial position on microcentrifuge tube, C. Ventral view of *H*. *vitripennis* head, D. Initiation of probing session, E. and F. probing and deposition of sheath saliva.

Two hundred *H*. *vitripennis* adults were collected from sweet orange and tangerines at the University of California Riverside Agricultural Operations in Riverside (Riverside County) California. They were transferred to sleeve-cages containing *X*. *f*. *piercei*-infected grapevines, and allowed to feed for a 48 hours acquisition access period. After 48 hours sharpshooters were transferred in groups of 30 to either sweet orange or grapevines and allowed to feed for a period of 48 hours. Three replicates of 30 *H*. *vitripennis* on each host were established with insects confined to host plants by sleeve cages which were constructed from fine mesh, 50 cm long and 15 cm in diameter with a string tie at both ends. Subsequently, sharpshooters were collected and starved in empty sleeve cages for about one hour to stimulate probing. Afterward, each sharpshooter was transferred into a sterile 1.5 ml microcentrifuge tube using a sterile forceps or by stimulating movement by lightly taping on the microcentrifuge tube, and allowing the sharpshooter to walk backwards out of the enclosure. A single probe session, indicated by the formation of saliva in the centrifuge tube was allowed. Deposition of saliva could be observed readily under a stereoscope (Figure 1. A-F). The site of salivation was marked and the sharpshooter was transferred to another sterile 1.5 ml microcentrifuge tube. This was continued for a maximum of five probes, each in a different tube for each sharpshooter.

Saliva from each probing site (= salivation site) was collected by placing 5 µl of sterile Phosphate buffered saline (PBS) with a sterile 10 µl pipette tip on the site and pipetting up and down. All of the liquid then was drawn into a pipette and placed in a clean 1.5 ml microcentrifuge tube for DNA extraction. DNA was extracted using Extract-N-Amp Kit (Sigma, www.sigmaaldrich.com). In this extraction method, 25 µl of Extraction Solution (Sigma, product code E7526) was added to the 5 µl saliva solution, vortexed briefly, and then incubated in a heating block at 95° C for 10 minutes. At the end of this period, samples were removed and 25 µl of the Dilution Solution (Sigma, product code D5688) was added to the tube. The mixture then was vortexed and stored at 20° C until analyzed.

Extraction of DNA also was done from *H*. *vitripennis* heads using the Qiagen Tissue Kit (Qiagen Inc., www.qiagen.com). The whole sharpshooter body was surface sterilized to eliminate possible contaminants. Surface sterilization involved placing sharpshooters in 75% EtOH for 2 minutes, transferring them to a container with 10% household bleach for two minutes, and then rinsing them twice in sterile double-distilled H2O. After the surface sterilization step, each sharpshooter was transferred into a sterile Petri dish and its head and eyes were removed using a sterile scalpel. The head, which contained the foregut, was removed, the eyes excised and the remainder of the head was then placed into a sterile 1.5 ml microcentrifuge tube containing 150 µl of sterile PBS. The mixture was macerated using an electric mortar and sterile plastic pestles.

Detection and quantification of bacterial titers was done using real-time quantitative PCR (qPCR)in a Rotor Gene 3000 (Corbett Research, Australia, www.corbettlifescience.com). The qPCR assay included primers and a probe specific for *X*. *f*. *piercei* 16S rDNA (Schaad et al. 2002); forward primer (XFF2–16s, 5′ CTCGCCACCCATGGTATTACTAC 3′), reverse primer (XFR2–16s, 5′ CTGGCGGCAGGCCTAAC 3′) and a TaqMan probe (XfP2, 5′ Quasar 670 ATGTGCTGCCGTCCGACTTGCATG BHQ,-2 3′). The qPCR assays were done in 0.1 ml strip-tubes (Corbett Research) with 10µl 1X IQ Supermix (BioRad, www.bio-rad.com) that included 100mM KCl, 40 mM Tris-HCL (pH 8.4), 1.6 mM dNTPs, iTaq DNA polymerase, 50 units/ml, and 6 mM MgC12. *X*. *f*. *piercei* 16s rDNA primers were added in a concentration of 100 nM and 200 nM of each forward and reverse primer, respectively. *X*. *f*. *piercei* TaqMan probe was added in a concentration of 100 nM. The qPCR master mix included 5.8 µl of PCR-grade water and 2 µl of DNA template for a total reaction volume of 20 µl. *X*. *f*. *piercei* titers present on each sharpshooter foregut were quantified by a qPCR assay that included five 10-fold dilution points ranging from 550000 to 5 copies/µl that served as standards. Each sample was tested in triplicate and each qPCR run included two non-template controls for reference.

**Table 1.**
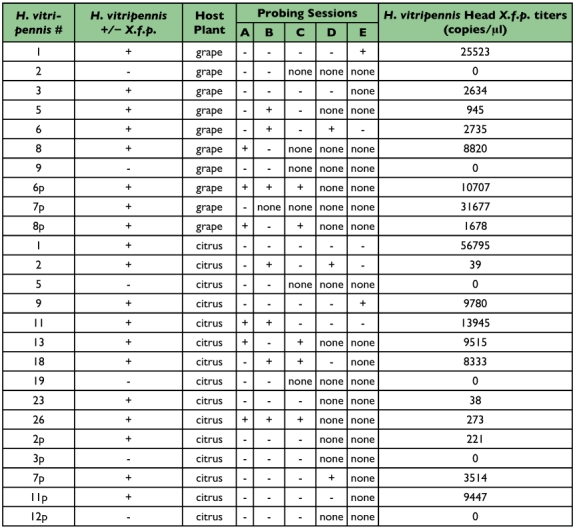
Representative data set obtained from *H*. *vitripennis* probing sessions. Field-collected *H*. *vitripennis* were confined to *X*. *fastidiosa*-infected grapevines for an acquisition access period of 48 h., transferred to either grapevines or citrus for 48 hours and then starved for 1 hour prior to their transfer into a sterile 1.5 µl microcentrifuge tube. Table details whether a sharpshooter was *X*. *f*. *piercei*-infected (+/-), type of post-acquisition plant host (grapevines or citrus), probing session (whether it was +/- or none = no successful probing session) and *X*. *f*. *piercei* titers estimated from each individual sharpshooter. *Xylella fastidiosa* detection and titer quantification was conducted by real-time PCR.

## Data Analysis

*H*. *vitripennis* were grouped according to *X*. *f*. *piercei* titer in their foregut: sharpshooters with *X*. *f*. *piercei* titers of less than 2500 copies/µl and those with titers of more than 2500 *X*. *f*. *piercei* copies/µl of sharpshooter head. This was done to see any effects of *X*. *f*. *piercei* titer on the frequency of *X*. *f*. *piercei* detection in *H*. *vitripennis* saliva. Proportion values were arc-sine transformed and used in a *t*-test. All statistical analyses were conducted using SPSS 10.0 software (SPSS Inc., Chicago). A *P*- value less than 0.05 was considered as statistically significant.

## Results and Discussion

### *Xylella fastidiosa* subsp. *piercei* titers in heads and saliva

*H*. *vitripennis* adults contained a broad range of *X*. *f*. *piercei* titers (from 2 to 32,000 copies/µl of *H*. *vitripennis* head, Table 1). Table 1 shows a representative data set obtained from the probing sessions indicating whether the sharpshooter head tested positive, the titers of *X*. *f*. *piercei* found in each individual and the number of positive or negative saliva samples from each probing session. *X*. *f*. *piercei* in saliva samples were found to be below 5 copies/ µl of saliva sample. Greater *X*. *f*. *piercei* acquisition (or greater *X*. *f*. *piercei* titer) did not result in more *X*. *f*. *piercei* positive saliva samples (*p* > 0.05, *t*-test, Table 2 and Table 3). The average frequency *X*. *f*. *piercei* detection in saliva from probing sites was 26% from *H*. *vitripennis* that fed on grapevines and 36% for those that fed on citrus. These two results were not statistically different (p > 0.05, *t*-test).

**Table 2.**
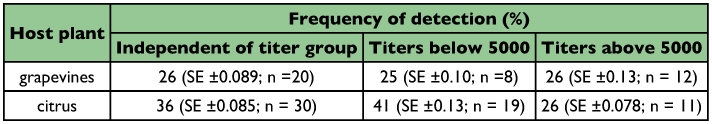
*Xylella fastidiosa* frequency of detection (%) in saliva samples from *H*. *vitripennis* that fed on grapevines or citrus. Table shows frequency of detection calculated independent of titer group and when the data set was partitioned in two groups containing below or above 5000 copies/µl of sharpshooter head.

**Table 3.**
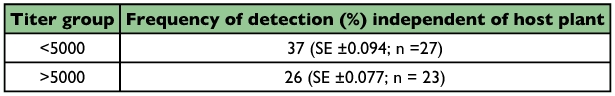
*Xylella fastidiosa* frequency of detection (%) in saliva samples from *H*. *vitripennis* that had *X*. *f*. *piercei* cells below and above 5000 cells in their foregut. Analysis is independent of host plant.

When the *H*. *vitripennis* estimated *X*. *f*. *piercei* titers were grouped in two main bacterial load groups (below 5000 copies/µl and above 5000 copies/µl), no difference was observed in the *X*. *f*. *piercei* detection frequency in saliva samples from *H*. *vitripennis* that fed on grapevines or citrus (*p* > 0.05, Table 2). Furthermore, no titer group differences were observed in the frequency of *X*. *f*. *piercei* detection within *H*. *vitripennis* that fed on grapevines (*p* > 0.05, Table 2) or within those that fed on citrus (p > 0.05, Table 2). The absence of any correlation of *X*. *f*. *piercei* titers to presence of this bacterium in the saliva of *H*. *vitripennis* is similar to what has been observed in transmission studies (Hill and Purcell 1995; Almeida and Purcell 2003). It has been suggested that as few as 100 *X*. *f*. *piercei* cells per sharpshooter head are sufficient for successful transmission (Purcell 2005). Further research is needed to assess the importance in transmission of these few cells (<5 copies/µl or less than 100 *X*. *f*. *piercei* cells per saliva sample) that are dislodged during the earlier stages of probing.

**Figure 2.**
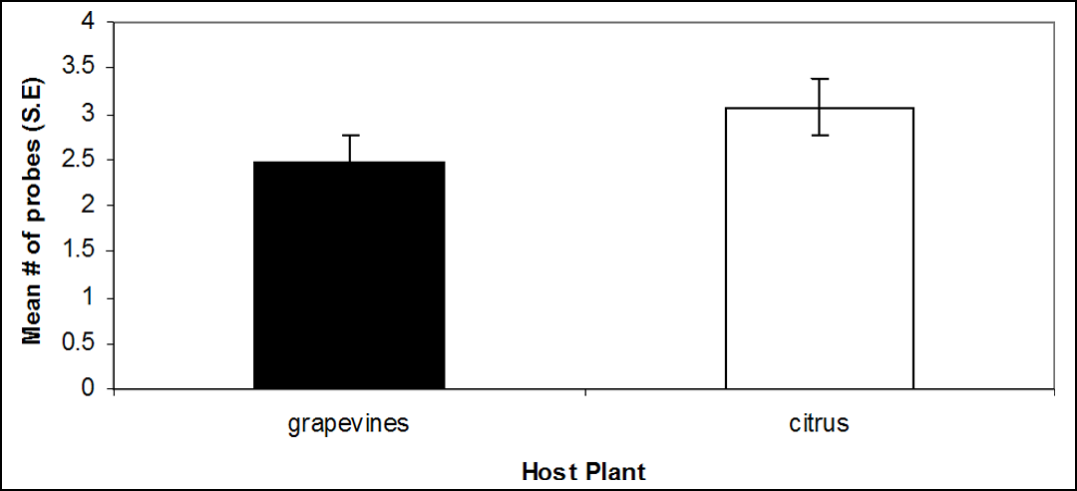
Probe frequency comparison between *H*. *vitripennis* that had grapevines or citrus as hosts. This graph represents probe sessions for all sharpshooters including those that tested negative for *X*. *f*. *piercei*.

**Video 1.** Glassy-winged sharpshooter probing attempts and deposition of saliva in lateral view. Observe *H*. *vitripennis* head movements and release of blobs of sheath saliva as it tries to probe through the plastic. This video can be accessed at the following URl: http://digital.library.wisc.edu/1793/24471

**Video 2.** Glassy-winged sharpshooter probing attempts and deposition of saliva in ventral view. Observe *H*. *vitripennis* saliva being deposited as it probes the surface of the microcentrifuge tube. This video can be accessed at the following URI: http://digital.library.wisc.edu/1793/24470

### Probing behavior

Young *H*. *vitripennis* adults appeared to probe more actively and more frequently than older adults (observation only). *H*. *vitripennis* adults on citrus were observed to be more active and to probe slightly more frequently than those on grapevines. This difference was not significant (*p* > 0.05; West, Figure 2).

Probing sessions started with the vertical positioning of the labium and a slight touch of the probing surface. This initial contact of the labial sensilla with the probing surface was followed by a full contact between the labium and the surface and the release of a small blob of sheath saliva (see Video 1 and Video 2). After this initial deposition of saliva, *H*. *vitripennis* either pulled the stylets back to the resting position or continued into a more prolonged probing. An active probing session included the sharpshooter positioning itself with the forelegs extended slightly further than when in rest. During the probing session the insect had five or six abrupt head movements that resulted in fluttering of the tip of the maxillae as described more fully in Backus et al. (2005) and Joost et al. (2006). This fluttering consisted of rapid movements of the maxillary stylets followed by the protrusion of the mandibular stylets and release of saliva.

Probing sessions lasted from three to 50 seconds. In some cases *H*. *vitripennis* adults probed for more than 3 minutes and on rare occasions some rested with their stylets on the plastic for more than 10 minutes. No differences were observed in the number of probing attempts of *H*. *vitripennis* that had grapevines or citrus as host plants (*p* > 0.05, *t*-test, Figure 2).

The use of this method to test for presence of *X*. *f*.. *piercei* in *H*. *vitripennis* saliva presents a novel approach for the study of pathogen movement during the initial stages of feeding. This method also allows an infectivity assessment of each individual sharpshooter without the need for sacrificing the insect and in a relatively clean environment. reducing the chances of cross-contamination that may occur with whole tissue extracts.
